# IL-21 Receptor Blockade Shifts the Follicular T Cell Balance and Reduces *De Novo* Donor-Specific Antibody Generation

**DOI:** 10.3389/fimmu.2021.661580

**Published:** 2021-04-09

**Authors:** Yeqi Nian, Zhilei Xiong, Panpan Zhan, Zhen Wang, Yang Xu, Jianghao Wei, Jie Zhao, Yingxin Fu

**Affiliations:** ^1^ Department of Kidney Transplantation, Tianjin First Central Hospital, School of Medicine, Nankai University, Tianjin, China; ^2^ Kidney Transplantation Research Laboratory, Tianjin First Central Hospital, Tianjin, China; ^3^ Key Laboratory of Transplantation, Chinese Academy of Medical Sciences, Tianjin, China; ^4^ Tianjin Key Laboratory for Organ Transplantation, Tianjin First Central Hospital, Tianjin, China

**Keywords:** follicular T helper cells, T-follicular regulatory cells, IL-21, donor specific antibodies, antibody mediated rejection, chronic rejection

## Abstract

Donor-specific antibodies (DSAs) play a key role in chronic kidney allograft injury. Follicular T helper (Tfh) cells trigger the humoral alloimmune response and promote DSA generation, while T-follicular regulatory (Tfr) cells inhibit antibody production by suppressing Tfh and B cells. Interleukin (IL)-21 exerts a distinct effect on Tfh and Tfr. Here, we studied whether blocking IL-21R with anti-IL-21R monoclonal antibody (αIL-21R) changes the Tfh/Tfr balance and inhibits DSA generation. First, we investigated the impact of αIL-21R on CD4^+^ T cell proliferation and apoptosis. The results showed that αIL-21R did not have cytotoxic effects on CD4^+^ T cells. Next, we examined Tfh and regulatory T cells (Tregs) in an *in vitro* conditioned culture model. Naïve CD4^+^ T cells were isolated from 3-month-old C57BL/6 mice and cultured in Tfh differentiation inducing conditions in presence of αIL-21R or isotype IgG and differentiation was evaluated by CXCR5 expression, a key Tfh marker. αIL-21R significantly inhibited Tfh differentiation. In contrast, under Treg differentiation conditions, FOXP3 expression was inhibited by IL-21. Notably, αIL-21R rescued IL-21-inhibited Treg differentiation. For *in vivo* investigation, a fully mismatched skin transplantation model was utilized to trigger the humoral alloimmune response. Consistently, flow cytometry revealed a reduced Tfh/Tfr ratio in recipients treated with αIL-21R. Germinal center response was evaluated by flow cytometry and lectin histochemistry. We observed that αIL-21R significantly inhibited germinal center reaction. Most importantly, DSA levels after transplantation were significantly inhibited by αIL-21R at different time points. In summary, our results demonstrate that αIL-21R shifts the Tfh/Tfr balance toward DSA inhibition. Therefore, αIL-21R may be a useful therapeutic agent to prevent chronic antibody mediated rejection after organ transplantation.

## Introduction

The clinical success of Tacrolimus-based immunosuppression, surgical techniques, and patient management has largely reduced short-term graft loss caused by acute rejection. However, improving long-term organ survival remains a challenge (1). The latest reports from the United States and Europe have shown almost no improvement in long-term kidney allograft survival during the last two decades (2, 3). Antibody-mediated rejection and the development of *de novo* donor-specific antibodies (dnDSAs) are recognized as distinct and common causes of late allograft injury and are responsible for one-third of failed allografts (4, 5). However, dnDSA generation is refractory to treatment with conventional immunosuppression, thus highlighting the need for a more comprehensive understanding of the underlying mechanism and the development of novel therapeutic strategies (6, 7).

T follicular helper (Tfh) cells are a distinct lineage of CD4^+^ T cells. Tfh cells are essential for mounting humoral immune response in secondary lymphoid organs, where they present help to B cells, form germinal centers (GCs), regulate affinity maturation, generate memory B cells, and activate long-lived plasma cells (8, 9). The function and presence of Tfh are linked to *de novo* DSA generation and chronic rejection (10). In contrast, T follicular regulatory (Tfr) cells, another CD4^+^ T cell type that express Forkhead Box P3 (FOXP3), suppress Tfh cell function, reduce GC reactions, and inhibit antibody responses (11). Notably, clinical data demonstrate that an increased Tfh/Tfr ratio is associated with antibody-mediated rejection and chronic renal allograft dysfunction (12, 13). Therefore, tilting the Tfh/Tfr balance toward inhibiting antibody production is a potential way to prevent chronic allograft injury.

Interleukin (IL)-21 is a critical cytokine in the humoral immune response and is primarily produced by Tfh cells. By binding to the IL-21 receptor (IL-21R), IL-21 promotes Tfh cell differentiation in an autocrine manner and induces its own expression (14). Notably, Tfh cell-mediated IL-21 functions are required for efficient antibody responses (15). Indeed, IL-21R-deficient patients showed compromised Tfh cell differentiation, reduced serum IgG levels, and poor antibody responses following vaccination with T cell-dependent antigens (16). Intriguingly, IL-21R exerts different effects in Tfr cells than in Tfh cells (17). In a mouse model, IL-21 reduced FOXP3 expression and influenced regulatory T (Treg) cell homeostasis (18). Moreover, Treg expansion was observed in patients with a loss-of-function IL-21R mutation (17). This demonstrates the distinct effect of IL-21 on Tfh and Tfr cells and suggests its potential as a target to modulate Tfh/Tfr balance.

In this study, we aimed to modulate the Tfh/Tfr balance using a monoclonal antibody against IL-21R (αIL-21R) and evaluated effects of αIL-21R on dnDSA generation. Our results show that αIL-21R shifts the Tfh/Tfr ratio to inhibit the humoral alloimmune response, thereby providing a novel way to prevent dnDSA generation.

## Method

### Animals

Wide-type male C57BL/6 (H2b, 8–12 weeks) and BALB/c (H2d, 8-12 weeks) mice were purchased from Vital River Laboratories (Beijing, China). Animals were allowed free access to water and standard chow. Animal experiments were approved by the Institutional Ethics committee for Research on Animals. The use and care of animals were in accordance with the Institutional Animal Care and Use Committee guidelines.

### αIL-21R

Monoclonal αIL-21R was obtained from BioXcell (Lebanon, NH, USA), diluted with PBS, and added to the cell culture medium at 10 μg/mL. For *in vivo* experiments, mice were treated with intraperitoneal injections of 1 mg/kg αIL-21R or isotype starting on the day of transplantation and three times per week for three weeks.

### Cell Isolation and T Cell Differentiation

Single cell suspensions were prepared from the spleens of C57BL/6 mice. Naive CD4^+^ T cells were isolated by negative selection, using biotinylated antibodies directed against markers of non-naive CD4^+^ T cells (CD8, CD11b, CD11c, CD19, CD24, CD25, CD44, CD45R, CD49b, TCRγ/δ, and TER119) and streptavidin-coated magnetic particles (Stemcell Technologies, Vancouver, BC, Canada). Cells were used at a purity of > 95%.

Naive CD4^+^ T cells were cultured in 96-well flat-bottom plates (2.0 x 10^5^ per well) suspended in 0.5 mL RPMI 1640 media supplemented with 10% fetal calf serum, 200 mM L-glutamine, 100 U/mL penicillin/streptomycin and 5 × 10^-5^ M 2-mercaptoethanol at 37°C in a 95%/5% air/CO_2_ atmosphere with saturating humidity. The wells were coated with 8 μg/mL anti-mouse α-CD3, and 2 μg/mL soluble anti-mouse α-CD28 was added to the media. The Tfh cell differentiation procedure was modified from Gao et al. (19) T cell-depleted splenocytes were stimulated with lipopolysaccharide (LPS) for 24 h and co-cultured with naive CD4^+^ T cells (1:10) to stimulate Tfh cell differentiation. IL-6 (100ng/mL) and IL-21 (50ng/mL) were added to the cell culture. For Treg differentiation, a cocktail containing IL-2, TGF-β, and all-trans retinoic acid (Immunocult; Stemcell Technologies, Vancouver, BC, Canada) was added to cell culture. After 5 days, the cells were collected, and analyzed by flow cytometry. To determine the absolute number of viable lymphocytes, Trypan blue staining was performed and live cells were counted using a hemocytometer.

### Flow Cytometry

Single cell suspensions were prepared from the spleens of recipient mice. Cells were labeled for surface and intracellular markers using fluorescence-labeled anti-mouse α-CD4, α-CXCR5, α-ICOS, α-PD1, α-FOXP3, α-GL7, and α-Fas antibodies (eBioscience, CA, USA). Intracellular FOXP3 staining was performed using a commercially available staining kit that included permeabilization solution and buffer (eBioscience). Flow cytometric measurements were performed on a FACSAria III (BD Bioscience, CA, USA) and data were analyzed using FlowJo (FlowJo Software, OR, USA). For proper gating, apoptotic cells were excluded, and the samples were compared with isotype controls, fluorescence minus one (FMO)-stained, permeabilized, and unpermeabilized unstained cells. For the carboxyfluorescein diacetate succinimidyl ester (CFSE; Invitrogen, CA, USA) cell proliferation assay, cells were labeled with 1 µM CFSE. The CFSE dilution was measured by flow cytometry.

### Skin Transplantation

Fully mismatched skin transplants were performed as described previously (20). In detail, full-thickness skin grafts (1 cm^2^) were obtained from the tails of BALB/c mice and engrafted onto the dorsolateral thoracic walls of C57BL/6 mice. Graft rejection was monitored daily and necrosis > 90% indicated graft rejection.

### Lectin Histochemistry

To analysis the germinal center, paraffin sections of spleen were deparaffinized, microwaved, immersed in 0.01 M citrate buffer (pH 6.0) for 20 min, washed with PBS, and incubated at room temperature for 30 minutes with biotinylated peanut agglutinin (PNA). After washing, tissues were incubated with peroxidase for 30 minutes. Reaction signals were developed using 3, 3’ diaminobenzidine (DAB; Dako, Denmark).

### Donor-Specific Antibody (DSA) Assay

For DSA detection, serum was diluted (1:18) and incubated with 10^6^ BALB/c splenocytes for 1 h at 4°C. Next, the cells were washed and incubated with anti-IgG and anti-IgM antibodies (Jackson immunoResearch, PA, USA) for 30 min. The mean fluorescence intensity of the cells was measured by flow cytometry.

### Statistical Analysis

Differences between groups were analyzed using unpaired Student’s *t*-tests. Graft survival was compared using log-rank tests. Results with p < 0.05 were considered statistically significant (*p<0.05; **p<0.01; ***p<0.001).

## Results

### αIL-21R Does Not Alter CD4^+^ T Cell Proliferation and Apoptosis Under Th0 Conditions

**Figure 1:**
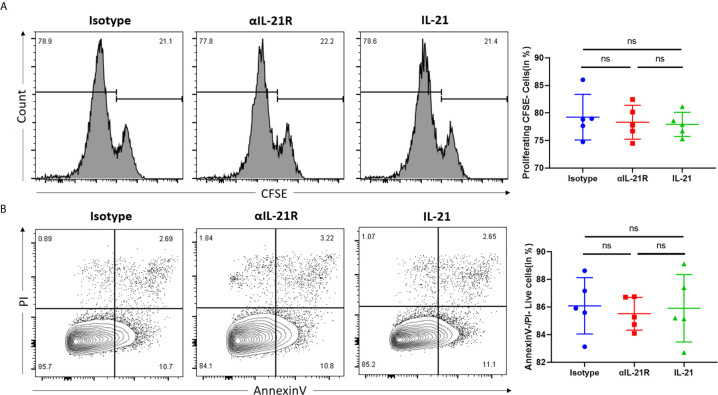
αIL-21R does not alter CD4+ T cell proliferation and apoptosis under Th0 conditions. **(A)** Naïve CD4+ T cells were isolated from C57BL/6 mice and labeled with 1μM CFSE. Naïve CD4+ T cells were activated with anti-CD3/anti-CD28 to induce proliferation. Proliferation of naïve CD4+ T cells was measured by the dilution of CFSE, in the presence of 10μg/ml αIL-21R or Isotype control. **(B)** Naïve CD4+ T cells from C57BL/6 mice were activated with anti-CD3/anti-CD28 under the condition of 10μg/ml αIL-21R or Isotype control. Lived cell was measured by the frequency of Annexin V^-^PI^-^ cells. Column plots display individual data points and mean ± SD, n = 5/group. ns, not significant.

To test the overall effect of αIL-21R on CD4^+^ T cells, we first investigated whether this monoclonal antibody induced proliferation or apoptosis in the unspecific Th0 condition. As shown by the CFSE proliferation assay, CD4^+^ T cells treated with αIL-21R or isotype control showed comparable proliferating cell rates (**Figure 1A**). Moreover, there was no significant difference the number of in live cells, dead cells, or apoptotic cells between the groups. These results indicate that αIL-21R is not cytotoxic and is not expected to kill a large number of cells or inhibit cell proliferation (**Figure 1B**).

### αIL-21R Inhibits Tfh Cell Differentiation and Rescues the IL-21 Induced Inhibition in Treg Cells

**Figure 2:**
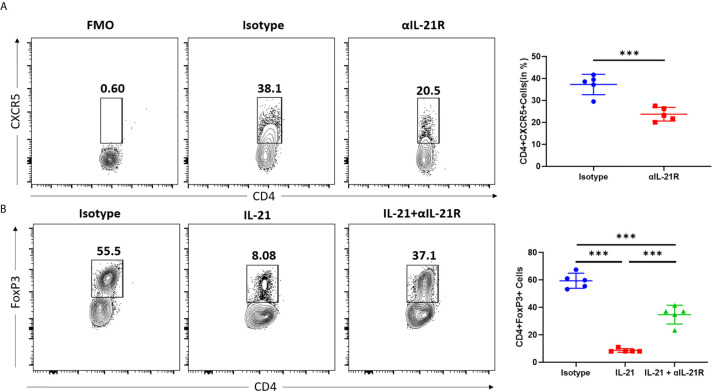
αIL-21R inhibits Tfh cell differentiation and rescues the IL-21 induced inhibition in Treg cells. **(A)** Naïve CD4+ T cells were isolated from C57BL/6 mouse and cultured under Tfh condition in the presence of 10μg/ml αIL-21R or Isotype control. Frequencies of CD4^+^CXCR5^+^ Tfh cells were analyzed by flow cytometry. **(B)** Naïve CD4^+^ T cells were isolated from C57BL/6 mouse and cultured under Treg condition, in the presence of 10ug/ml IL21, with αIL-21R or Isotype control. Frequencies of CD4^+^FOXP3^+^ Treg cells were analyzed by flow cytometry. Column plots display individual data points and mean ± SD, n = 5/group. Fluorescence minus one (FMO) controls are the experimental cells stained with all the fluorophores minus the fluorophore of target. FMO controls were used to properly interpret flow cytometry data. ***p<0.001.

Both Tfh and Tfr cells expressed IL-21R. However, IL-21 promoted Tfh differentiation and inhibited the expression of FOXP3, the signature transcription factor of Tfr and Treg cells. Furthermore, αIL-21R decreased the Tfh/Tfr ratio by inhibiting Tfh differentiation while blocking the IL-21 induced inhibition of Tfr cells. To assess the effect of αIL-21R on Tfh/Tfr ratio, we individually examined the impact of αIL-21R on Tfh and Treg differentiation. Under Tfh differentiation conditions, the frequency of Tfh cells reduced significantly by αIL-21R treatment (**Figure 2A**). Since a well-established method for Tfr differentiation is lacking, we observed the effect of αIL-21R on Tregs. As shown in **Figure 2B**, IL-21 reduced the frequency of Treg cells, and this effect was reversed by αIL-21R treatment (**Figure 2B**). Notably, αIL-21R treatment alone did not alter Treg differentiation (data not shown). Taken together, these data suggest that αIL-21R treatment can modify the Tfh/Tfr balance.

### αIL-21R Decreases the Tfh/Tfr Ratio in Transplant Recipient Mice

**Figure 3:**
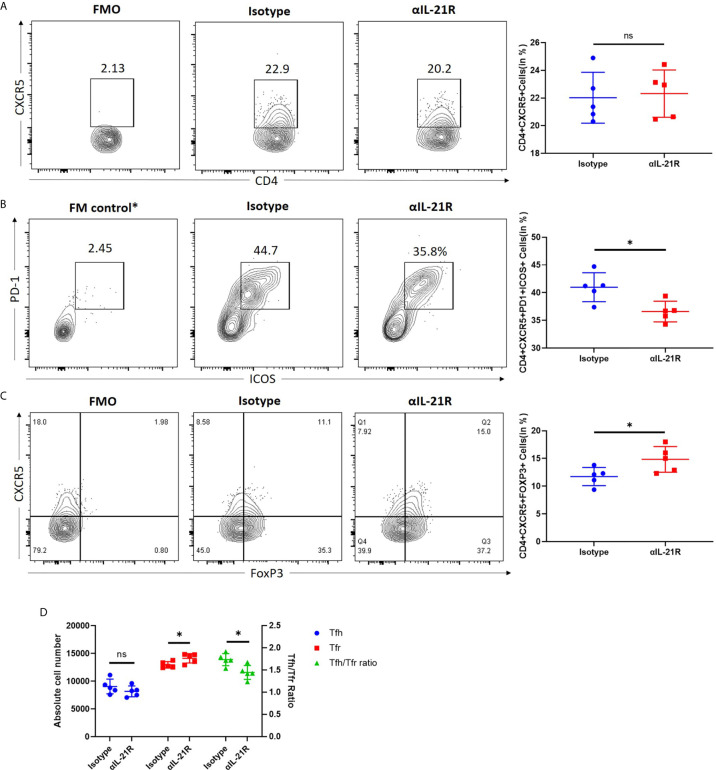
αIL-21R decreases the Tfh/Tfr ratio in transplant recipient mice. Skin allografts from BALB/c mice were transplanted onto C57BL/6 mice. Recipients were treated with 1 mg/kg αIL-21R three times every week or Isotype starting on the day of transplantation. **(A)** Frequencies of CD4^+^CXCR5^+^ T cells and **(B)** frequencies of CD4^+^CXCR5^+^ICOS^+^PD1^+^ Tfh cells and **(C)** frequencies of CD4^+^CXCR5^+^FOXP3^+^ Tfr cells in the spleen were assessed after 14 days by flow cytometry gating on single cells. **(D)** Absolute number of Tfh, Tfr cells and Tfh/Tfr ratio were calculated based on the results of flow cytometry by multiplication total number per sample and percentage of interested cells. Column plots display individual data points and mean ± SD, n = 5/group. *p<0.05.

A skin transplantation model was used to investigate the effect of αIL-21R treatment on the Tfh/Tfr ratio in the context of alloimmune responses. Mice that received fully mismatched skin allografts were treated with αIL-21R or isotype control for 14 days after surgery and their splenocytes were analyzed. We observed that αIL-21R treatment did not reduce the frequency of CD4^+^CXCR5^+^ T cells (**Figure 3A**). However, a significant decrease was observed in the frequency of CD4^+^CXCR5^+^ ICOS^+^PD^+^ Tfh cells (**Figure 3B**). Meanwhile, αIL-21R increased the frequency of CD4^+^CXCR5^+^FOXP3^+^ Tfr cells (**Figure 3C**). The Tfh/Tfr ratio was calculated as the absolute number of CD4^+^CXCR5^+^PD1^+^ICOS^+^ and CD4^+^CXCR5^+^FOXP3^+^ cells. The results showed that αIL-21R significantly decreased the Tfh/Tfr ratio (**Figure 3D**). Notably, the absolute number of Tfh cells was lower in the group treated with αIL-21R, although the results was statistically insignificant (**Figure 3D**).

### GC Reaction and dnDSA Generation Are Blocked by αIL-21R

**Figure 4:**
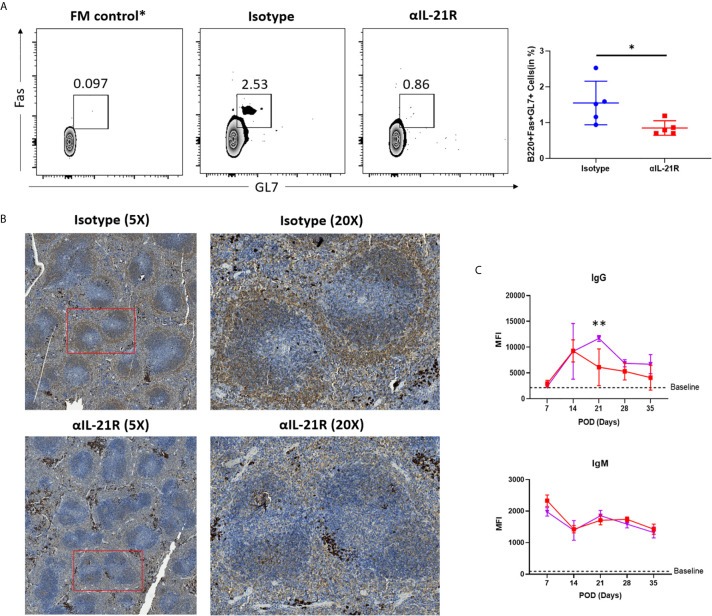
GC reaction and dnDSA generation are blocked by αIL-21R. Skin allografts from BALB/c mice were transplanted onto C57BL/6 mice. Recipients were treated with 1 mg/kg αIL-21R three times every week or Isotype starting on the day of transplantation. **(A)** Frequencies of B220^+^GL7^+^Fas^+^ GC B cells were assessed after 14 days by flow cytometry. **(B)** Spleens were harvest from recipients after 14 days of transplantation and stained with PNA. GC was identified as brown area in while pulp. **(C)** Serum was collected from recipients every week after transplantation. IgG and IgM level were detected with flow cytometry. Column plots display individual data points and mean ± SD, n = 5/group. *p<0.05, **p<0.01.

To assess whether the *in vivo* decrease in Tfh/Tfr ratio mediated by αIL-21R inhibited the antibody response, we treated skin transplantation recipients with αIL-21R or control IgG and analyzed the GC response after 14 days. Flow cytometry analysis revealed a significant decrease in the frequency of GC B cells (B220^+^GL7^+^Fas^+^ cells) in mice treated with αIL-21R (**Figure 4A**). In line with this finding, PNA staining confirmed that αIL-21R inhibited GC formation (**Figure 4B**). Finally, we investigated the effect of αIL-21R on dnDSA generation by monitoring the levels of donor-specific IgG and IgM for 5-weeks (**Figure 4C**). As previously reported, IgG levels in the control group continued to increase and peaked at 3-weeks post-surgery, after which, the IgG level decreased, and remained at a stable level. No significant difference was observed between the treatment and control groups during the first 2-weeks. However, the difference appeared at 2-weeks as αIL-21R treatment blocked increasing IgG levels, and even caused them to decrease. At 3-weeks after surgery, IgG levels in the αIL-21R treatment group were significantly lower than in the control group. Although no significant difference was observed afterward, the αIL-21R group showed an overall lower IgG level compared to the control group; no significant difference was observed in IgM levels between the groups (**Figure 4C**).

## Discussion

In this study, we evaluated the ability of αIL-21R to modulate the Tfh/Tfr balance and evaluated its effects on dnDSA generation. Our main finding from this study is that αIL-21R shifts the Tfh/Tfr ratio toward inhibition of humoral immune response and reduces dnDSA generation against a fully mismatched allograft. Furthermore, the IgG kinetics observed in the control group were consistent with the natural course of allosensitization (21). The IgG level continued to increase after transplantation and peaked at 3-weeks, after which it decreased, and remained at a stable level; however, this level was still higher than the baseline level. In contrast, αIL-21R treatment significantly altered the IgG generation kinetics. Indeed, the time of IgG increase was shorter and peak was lower. Given that earlier clinical data demonstrated the association between dnDSA and chronic allograft injury, our results provide new evidence to support the use of αIL-21R in organ transplantation to improve long-term outcomes (4, 5).

Notably, the kinetics observed in this study are in line with the Tfh/Tfr dynamic balance observed in the humoral response. Tfr cells control antigen-specific Tfh cell expansion and terminate the GC reaction (22). In the B cell follicle, Tfh and Tfr cells are present in an equal proportion (23). Upon antigen challenge, both Tfh and Tfr cells begin to expand (24) but they do not proliferate at a similar rate. Tfh cells proliferate faster and shift the Tfh/Tfr ratio in favor of helper T cell generation. Indeed, the Tfh cell response reached its peak at postoperative day 7, while Tfr cells represented a far lower proportion of the follicular CD4^+^ T cell population. GCs begin to form at this time point and start to generate antibodies. By day 10, the Tfh numbers started to decrease, while Tfr cells continued to proliferate. Thus, the Tfh/Tfr ratio shifted toward inducing termination of the GC reaction. The mean Tfh/Tfr ratio was 1.44 and the DSA continued to increase until day 21. In contrast, the mean Tfh/Tfr ratio was 1.73 in the αIL-21R treatment group and DSA began to decrease on day 14. Thus, modulating the Tfr/Tfr ratio with αIL-21R is a potential strategy to limit dnDSA generation and may have applications in clinical settings in the future.

The mechanism underlying the αIL-21R effect is to modulate the differential effect of IL-21 on Tfh and Tfr cells. Antigen-stimulated Tfh cells proliferate rapidly and produce IL-21 as a major effector cytokine. IL-21R is broadly expressed on T, B, natural killer, and dendritic cells. Further, IL-21 signaling, *via* Jak-Stat and other pathways, promotes their proliferation, differentiation, and effector function (25). However, in our study, IL-21 reduced the expression of FOXP3, the key transcription factor in Tfr cells. IL-2 is an essential cytokine for the expression of FOXP3. Webster et al. reported that IL-21 inhibits Tfr differentiation by inhibiting their response to IL-2 in a Bcl6-dependent mechanism (17). At the molecular level, Jak-STAT3 signaling is activated by IL-21 and leads to STAT1 and STAT3 phosphorylation, whereas IL-2 strongly activates STAT5. Thus, the ability of different STAT molecules to compete for STAT binding sites and affect gene transcription may underlie the differential effects of IL-21 on Tfh and Tfr (26, 27). In addition, Foxp3+ regulatory T cells suppress the emergence of long-lived splenic plasma cells, thus increased Foxp3 expression by blocking IL-21 receptor may diminish the DSA production (28).

In summary, this study reports αIL-21R mediated modulation of the Tfh/Tfr ratio and subsequent reduction in dnDSA generation. Given that IL-21R is broadly expressed, targeting IL-21 signaling may have multiple-target effects on the humoral immune response. Indeed, IL-21R antagonists inhibit the B cell differentiation toward plasmablasts when exposed to alloantigens (29). Despite the fact that dnDSA plays a central role in antibody-mediated chronic graft rejection, the lack of graft survival data is a major limitation of our study. However, our study provides a novel strategy to control chronic allograft injury. In further studies, IL-21R transgenic mice and a minor-mismatch heart transplantation model will be used to provide direct evidence that targeting IL-21R protects against chronic allograft injury.

## Data Availability Statement

The raw data supporting the conclusions of this article will be made available by the authors, without undue reservation.

## Ethics Statement

The animal study was reviewed and approved by Tianjin First Central Hospital, Nankai University.

## Author Contributions

YN and ZX performed experiments, analyzed data, and wrote the manuscript. PZ, YX, and JW supported experiments and edited the manuscript. JZ and YF designed experiments, supervised the work, and wrote the manuscript. All authors contributed to the article and approved the submitted version.

## Funding

This work was supported by a grant (81970654) from National Natural Science Foundation of China to YF and a grant (CM201812) from Tianjin First Center Hospital to JZ.

## Conflict of Interest

The authors declare that the research was conducted in the absence of any commercial or financial relationships that could be construed as a potential conflict of interest.
